# Seasonal Turnover in Bat Skin Mycobiota: Contrasting Fungal Communities Between Hibernation and Reproduction in Greater Mouse-Eared Bats (*Myotis myotis*)

**DOI:** 10.3390/pathogens15010083

**Published:** 2026-01-12

**Authors:** Rafał Ogórek, Jakub Suchodolski, Justyna Borzęcka, Tomasz Kokurewicz

**Affiliations:** 1 Department of Mycology and Genetics, Faculty of Biological Sciences, University of Wrocław, Przybyszewskiego Street 63-77, 51-148 Wrocław, Poland; justyna.borzecka@uwr.edu.pl; 2 Department of Vertebrate Ecology and Paleontology, Institute of Biology, Wrocław University of Environmental and Life Sciences, Kożuchowska 5b, 51-631 Wrocław, Poland; tomasz.kokurewicz@upwr.edu.pl

**Keywords:** skin mycobiota, bats, winter, summer, Poland

## Abstract

The skin of bats hosts diverse microbial communities, yet most research has focused on bacteria or single fungal pathogens such as *Pseudogymnoascus destructans*. Here, we present the first direct comparison of culturable skin mycobiota in the greater mouse-eared bat (*Myotis myotis*) between hibernation and the reproductive season. Swabs collected from hibernating bats in the Nietoperek reserve and from maternity colonies in Lipy yielded 41 fungal species, including 27 that represent new records for *M. myotis*. Winter assemblages were less diverse but strongly dominated by *Penicillium* (>90% of isolates), while summer maternity roosts supported broader communities shaped by environmental exposure and plant-associated fungi. Despite seasonal turnover, a small set of taxa, including *Aspergillus fumigatus*, *Mucor fragilis*, and *Pseudogymnoascus pannorum*, persisted across both seasons, indicating the presence of a limited core mycobiota. Richness was higher on wing membranes than on tail membranes, whereas biometric variables such as sex, age, body mass, and forearm length showed only weak and inconsistent associations with fungal diversity. These findings demonstrate that seasonal filtering is likely one of the main factors determining the skin mycobiota in *M. myotis*. Additionally, we expand the known fungal diversity of this species, and emphasize its role as a reservoir of environmental, opportunistic, and pathogenic fungi.

## 1. Introduction

Bats (*Chiroptera*) represent one of the most diverse groups of mammals and play an essential role in tropical ecosystems as pollinators and seed dispersers, while in temperate zones, insectivorous bats feeding on vast numbers agricultural and forestry pests are successfully suppressing their number [1,2]. Their annual cycle exposes them to different microclimatic conditions: during hibernation, they inhabit underground sites with relatively stable, low temperatures and high humidity, whereas in summer, they often form maternity colonies in attics or tree hollows, where conditions are warmer, less humid, and more variable [3,4]. These contrasting environments may strongly influence the composition and dynamics of the microbial communities associated with bats, especially fungi [5].

Fungal communities on the skin and wing membranes of bats are of particular importance. On one hand, they may include saprophytic or commensal species that form part of the natural microbiota; on the other, they can harbour opportunistic pathogens of medical and veterinary importance [6,7]. Moreover, certain cold-adapted fungi such as *Pseudogymnoascus destructans* are recognized as causative agents of white-nose syndrome (WNS), a devastating disease that has severely reduced bat populations in North America [8]. In Europe, where *Myotis myotis* remains relatively abundant, studies have shown that fungal diversity on these bats differs depending on season and microhabitat [7,9,10], but simultaneous comparisons between hibernation and maternity periods remain scarce. Our recent autumn data on *M. myotis* [7] showed that the skin mycobiota of this species differs between body regions and is influenced by host traits. We demonstrated higher fungal diversity on wing membranes than on tail membranes and reported age- and sex-related differences in fungal richness. We indicated that both microhabitat and host condition may affect the skin mycobiota during the autumn activity period. However, it is still unknown whether these patterns persist across the two strongest seasonal contrasts in the bats’ annual cycle, namely hibernation and the maternity period [7].

Temperature is one of the key factors structuring fungal communities. Stable, cold cave environments promote the growth of psychrophilic and psychrotolerant taxa, while fluctuating and often warmer conditions in summer roosts may favour mesophilic or thermotolerant species [11,12,13,14]. Such environmental contrasts are expected to shape both the diversity and the functional potential of bat-associated fungi, yet few studies have examined them simultaneously.

In light of these considerations, the present study aims to compare the cultivable fungal diversity associated with *M. myotis* wing and tail membranes during two critical stages of their annual cycle: the end of hibernation in a cold, stable underground environment and the reproductive period in a maternity colony exposed to fluctuating temperatures and lower humidity. By isolating fungi under three incubation conditions (5 °C, 24 °C, and 37 °C), we aimed to capture a broad spectrum of taxa with different thermal preferences. Furthermore, by integrating biometric parameters of the bats, we explored potential host-related factors influencing fungal colonization. Such an approach allowed the understanding of how seasonal, environmental, and biometric variation shapes bat-associated mycobiota.

## 2. Materials and Methods

### 2.1. Study Area

The study was conducted in the underground tunnels of the Nietoperek bat reserve (52°25′ N, 15°32′ E) during hibernation on 8 April 2021 (Section 7.2, from location nos. 4 to 6 in Borzęcka et al. [10]) and from a maternity colony in the attic of the forester’s lodge in Lipy on 31 August 2021 (52°52′ N, 15°17′ E). Both research locations are located in Western Poland in a ca. 60 km straight-line distance from each other. The first one is the largest bat hibernation site in Poland and one of the ten-largest in the European Union protected in November 2007 as the Natura 2000 site “Nietoperek” (area code: PLH080003). Out of 12 bat species found hibernating there, four, including *M. myotis*, are mentioned in Annex II of the European Union Habitat Directive (92/43/EEC) [15].

We do not have direct proof that the maternity colony in the attic of the forester’s lodge in Lipy is formed by the same individuals that hibernate in the Nietoperek bat reserve. However, taking into account our own data based on migrations of ringed individuals [16,17], proving the migration between summer and winter colonies exceeding 226.7 km, and taking the knowledge about the migration behaviour of *M. myotis* [18] and also the short distance between our study sites, we may assume with high certainty that the investigated individuals belong to the same population using Nietoperek underground tunnels as central winter roosts for individuals from nearby breeding colonies.

The study was performed according to the ARRIVE guidelines 2.0 [19]. Samplings in the Nietoperek bat reserve were made under licence no. WPN-I.6205.24.2021.MG issued by the Regional Directorate for Environmental Protection in Gorzów Wielkopolski (approval date: 29 March 2021), while that in the maternity colony in Lipy were made under licence no. DZP-WG.6401.179.2021.EB issued by the General Directorate for Environmental Protection in Warsaw (approval date: 3 August 2021).

### 2.2. Microclimatic Parameter Measurement

The air temperature (*T*a) and relative humidity (RH) at both sites were measured by loggers (i-button DS 1923, Texas Instruments, Dallas, TX, USA) recording microclimatic parameters at 10 min intervals with an accuracy to 0.1 °C and 0.6% on the day of sampling the fungi from captured bats.

### 2.3. Sampling Methods

Bats were collected manually in winter and summer colonies—30 bats from each location. To avoid contamination, immediately after capture, four body swabs were collected from each individual for further mycological analyses according to Borzęcka et al. [7]. Next, species [20], sex, age, reproductive status, forearm length (±0.05 mm accuracy), and body weight (Pesola balance, ±0.1 g accuracy) were recorded for each individual. Age was assessed non-invasively based on ossification of finger epiphyseal joints: fully ossified in adults and partially ossified with a visible gap in juveniles and subadults [21]. By late summer and autumn, juveniles develop stronger ossification and are classified as subadults; thus, two age categories were distinguished: adults and subadults.

Wing membrane swabs were collected using sterile, saline-moistened swabs (0.85% NaCl) in transport tubes (plastic applicator with 15 cm viscose swab) following Ogórek et al. [9]. Each bat was sampled with four swabs: two per wing (ventral and dorsal surfaces, covering the plagiopatagium, dactylopatagium, and propatagium) and two from the tail membrane (ventral and dorsal surfaces). The procedure lasted up to 15 min, after which bats were released at the capture site [9].

To minimize cross-contamination, the following precautions were applied: wearing surgical gowns and changing gloves between animals [7]. Samples were transported under cooling conditions (10 ± 2 °C), stored at 5 ± 0.5 °C, and processed within seven days [9]. Each swab was labelled with bat ID, membrane type, collection date, sex, and location. In total, 240 swabs were obtained from 60 bats.

### 2.4. Isolation of Fungi from Samples

Fungal isolation was performed using standard culture methods. Swabs were immersed in sterile 50 mL polypropylene tubes (FL Medical, Via Enrico Fermi, Italy) containing 3 mL of sterile distilled water and vortexed at room temperature (3 min, 500 rpm). Aliquots of 100 µL and 1000 µL were spread, in triplicate, onto potato dextrose agar (PDA; BioMaxima, Lublin, Poland). Plates were incubated in the dark at 5 ± 0.5 °C, 24 ± 0.5 °C, and 37 ± 0.5 °C for 5–90 days. The incubation temperatures represented (i) 5 °C, approximating hibernation site conditions and favouring psychrophilic conditions, monitored in the manner of Vanderwolf et al. [22]; (ii) 24 °C, optimal for most fungal species; and (iii) 37 °C, reflecting mammalian body temperature.

Pure cultures were obtained using the single hyphal tip method [23] and maintained on PDA slants at 5 or 24 ± 0.5 °C for molecular analyses and further characterization.

### 2.5. Identification of Fungi

Fungal identification combined phenotypic and molecular approaches. Macroscopic traits were evaluated on PDA, and for selected genera (*Aspergillus* or *Penicillium*), additional media were used: Czapek yeast autolysate agar (CYA), Czapek-Dox agar (1.2% agar; BioMaxima, Lublin, Poland), and malt extract agar (MEA; BioMaxima, Lublin, Poland) [9]. Colony growth rate, texture, pigmentation, sporulation, cleistothecia formation, production of soluble pigments and exudates, and colony reverse coloration were recorded. Microscopic traits included hyphal structures and spore morphology, observed in preparations from PDA and MEA cultures [23]. Identification was supported by diagnostic keys and standard mycological monographs [8,24,25,26,27,28,29,30,31,32].

Molecular confirmation involved sequencing of the rDNA internal transcribed spacer (ITS) region. For isolates not conclusively identified, partial β-tubulin gene sequencing was performed. Genomic DNA was extracted from 20-day-old PDA cultures using the Bead-Beat Micro AX Gravity kit (A&A Biotechnology, Gdańsk, Poland), following the manufacturer’s instructions. DNA quality was verified by electrophoresis on 1.2% agarose gels and by spectrophotometry (NanoPhotometer^®^ NP80, Implen, Munich, Germany).

The ITS region was amplified with primers ITS1 (5′-TCCGTAGGTGAACCTGCGG-3′) and ITS4 (5′-TCCTCCGCTTATTGATATGC-3′) [33], and in cases of inconclusive results, Bt2a_F (5′-GGTAACCAAATCGGTGCTGCTTTC-3′) and Bt2a_R (5′-ACCCTCAGTGTAGTGACCCTTGGC-3′) were applied [34]. PCRs were performed in a T100 Thermal Cycler (Bio-Rad, Berkeley, CA, USA) according to Ogórek et al. [11]. Amplification of DNA was performed in a 50 μL reaction mixture using the 2 × PCR mixture containing *Taq* polymerase (0.1 U µL^−1^), dNTP mix (2 mM), MgCl_2_ (4 mM) (Blirt), 0.25 μM of each primer, and 45 ng of genomic DNA. Products were verified by agarose gel electrophoresis, purified with the Clean-Up kit (A&A Biotechnology, Gdańsk, Poland), and sequenced by Macrogen Europe (Amsterdam, The Netherlands) using Sanger sequencing.

### 2.6. Data Analyses

Sequences were analyzed using the BioEdit Sequence Alignment Editor. Fungal ITS sequences were compared with GenBank records using the BLAST algorithm (BLAST+ 2.17.0, http://www.ncbi.nlm.nih.gov/, accessed on 2 September 2025), and the obtained sequences were deposited in GenBank. Interpretation followed the criteria of Zhang et al. [35].

Associations between fungal species richness and host traits (age, sex, forearm length, body mass) were assessed using Spearman’s rank correlation coefficient (r_S_) at α = 0.05. For analyses, age and sex were treated as binary variables (age: 0 = subadult, 1 = adult; sex: 0 = female, 1 = male) according to Borzęcka et al. [7].

## 3. Results

In total, 60 individuals of *M. myotis* were examined, i.e., 30 individuals from each study period. Of these, males dominated during hibernation (17 out of 30) and females dominated in maternity roosts (25 out of 30). Most of the individuals examined in the Nietoperek bat reserve were adults (18 out of 30), while in the attic of the forester’s lodge in Lipy, most of the individuals were subadults (28 out of 30) (Table A1).

### 3.1. Biometric Features of Bats and Microclimatic Conditions in Habitats

The body weight of *M. myotis* ranged from 22.0 g to 28.0 g (mean: 24.9 g ± 1.5), and their forearm lengths ranged from 58.7 mm to 64.1 mm (mean: 61.0 mm ± 1.4) during hibernation. In turn, the body weight of *M. myotis* ranged from 20.0 g to 29.0 g (mean: 24.6 g ± 2.0), and their forearm lengths ranged from 56.8 mm to 64.8 mm (mean: 61.7 mm ± 1.8) in maternity roosts (Table A1).

The microclimatic conditions at the sampling site in the Nietoperek underground tunnels ranged from 8.9 to 9.7 °C and 81 to 84%, while in the attic in Lipy, it ranged from 20 to 25 °C and 20 to 40%.

### 3.2. Fungal Isolation and Identification

Classical mycological analysis of the fungal isolates allowed for their classification into 22 groups (isolates UWR_744-UWR_765) from the Nietoperek bat reserve and 29 groups (isolates UWR_766-UWR_794) from the forester’s lodge in Lipy. These fungal groups were differentiated in macroscopic and/or microscopic colony morphology. Molecular analyses confirmed the species identity of the fungi from each group, allowing for verification of the initial phenotypic classification. However, in several cases, in addition to standard rDNA ITS region sequencing, β-tubulin gene sequencing was necessary to ensure effective species identification in the case of 10 isolates (Table 1).

Consequently, 22 and 29 culturable species of fungi were identified from bats during hibernation and from maternity roosts, respectively. These fungi represented two morphological forms: filamentous fungi (most species) and yeast-like fungi (*Aureobasidium pullulans*). Based on BLAST analysis, all fungal sequences had an E-value of zero and 100% query cover, except for isolate UWR_778. The sequence lengths ranged from 357 to 529 bp, and an identity range of 98.20–100% (Table 1).

### 3.3. Fungal Diversity Across Body Regions and the Effect of Age and Sex

**Figure 1:**
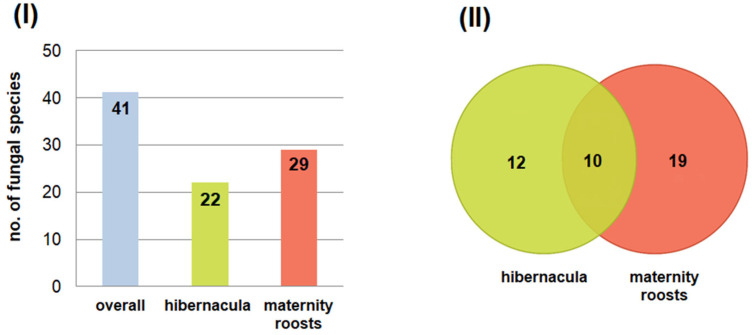
The number of cultured fungal species from the wing and tail membranes of *M. myotis* in the underground tunnels of the Nietoperek bat reserve during hibernation and in maternity roosts in the attic of the forester’s lodge in Lipy: (**I**) the number of species obtained at a given study site, and (**II**) the relationships between study sites and isolated species.

A total of 41 fungal species were detected across all sampled *M. myotis*, where 22 species were found on the skin of these small mammals in the underground tunnels of the Nietoperek bat reserve during hibernation and 29 were found in maternity roosts in the attic of the forester’s lodge in Lipy. Some fungal species were strictly associated with a specific habitat (12 species occurred only on bats during hibernation and 19 occurred in maternity roosts) and 10 species occurred in both studied locations (Figure 1).

**Figure 2:**
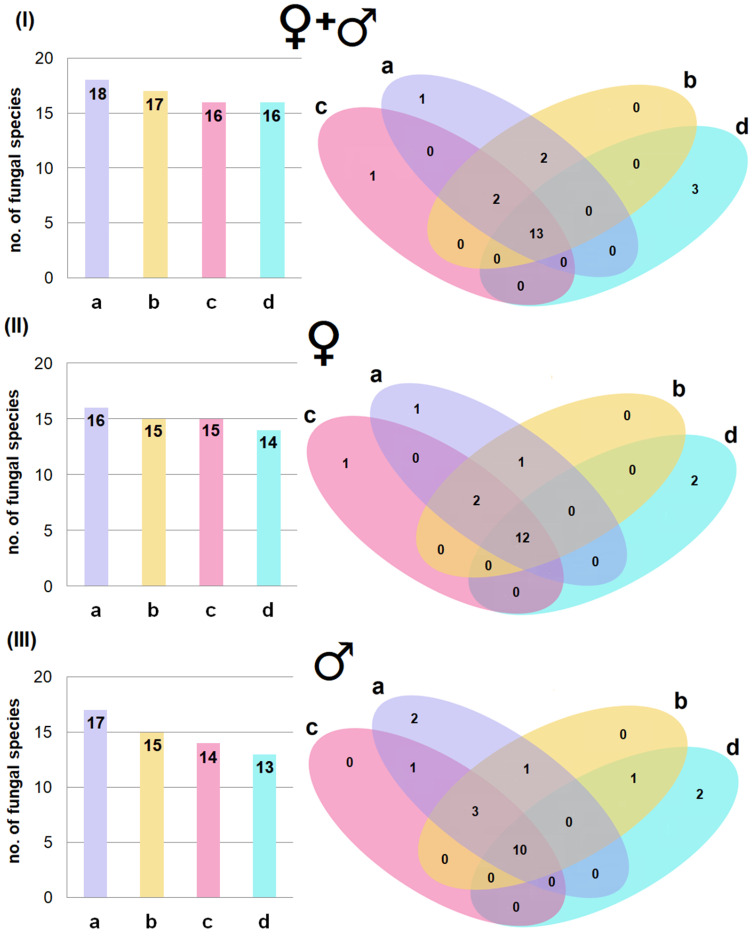
The number of fungal species isolated from the ventral (a) and dorsal (b) sides of the wing membranes, and the ventral (c) and dorsal (d) sides of tail membranes of *M. myotis* in the underground tunnels of the Nietoperek bat reserve during hibernation: (**I**) from all studied bats, (**II**) only from females, and (**III**) only from males.

Overall, 18 fungal species were found on the ventral side of the wing membranes in hibernating bats and 17 on the dorsal side. Additionally, 16 species were found on either the ventral side or on the dorsal side of their tail membranes (Figure 2I). A similar trend in the number of species inhabiting individual membranes was also observed when classifying hibernating bats based by sex. Thus, their wing membranes (especially the ventral side) harboured a more diverse fungal community than the tail membranes (Figure 2).

Moreover, some fungal species were strictly associated with specific body regions, with the highest number found on the dorsal side of tail membranes across all bats (Figure 2I: 3 species) and when divided by sex (Figure 2II: 2 species in females; Figure 2III: 2 fungal species in male). However, it should be noted that most fungal species were present across multiple body regions (Figure 2).

**Figure 3:**
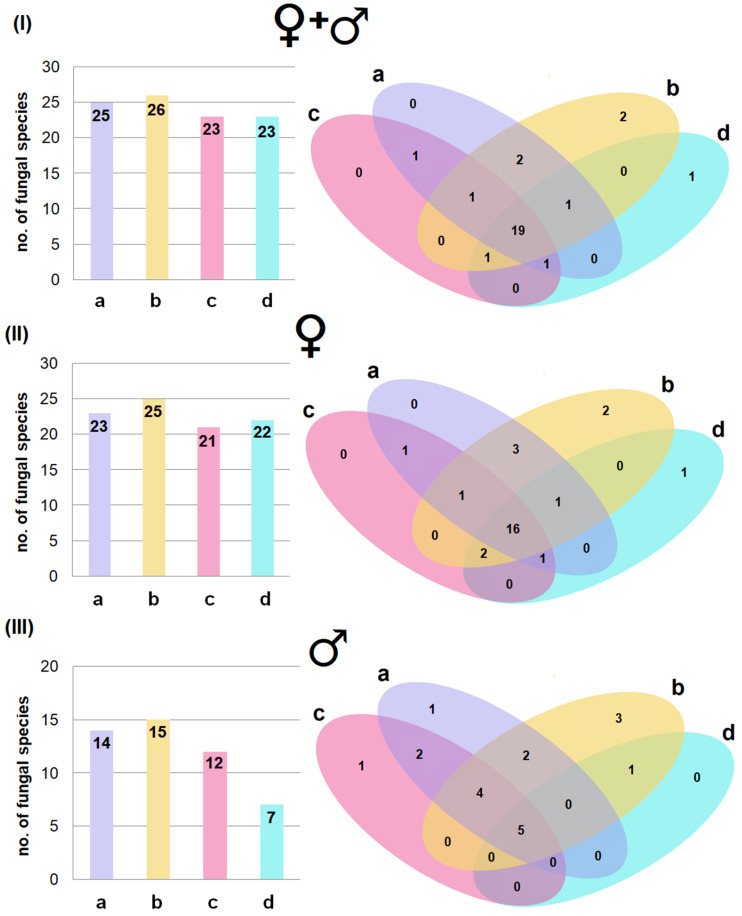
The number of fungal species isolated from the ventral (a) and dorsal (b) sides of the wing membranes, and the ventral (c) and dorsal (d) sides of tail membranes of *M. myotis* in maternity roosts in the attic of the forester’s lodge in Lipy: (**I**) from all studied bats, (**II**) only from females, and (**III**) only from males.

The wing membranes of bats in maternity roosts, similarly to those of hibernating individuals, harboured a more diverse fungal community than the tail membranes. However, in this case, the largest number of species was found for the dorsal side—26 fungal species for all bats, 25 for females and 15 species for males (Figure 3).

Most fungal species were present across multiple body regions (Figure 3), but it was also noted, as in the case of hibernating bats, that some fungal species found on the skin of bats in maternity roosts were closely associated with specific body areas. However, in this case, the largest numbers were found on the dorsal sides of the wing membranes, both in all bats (Figure 3I: two fungal species) and when divided by sex (Figure 3II: two fungal species for females; Figure 3III: three fungal species for males).

### 3.4. Effect of Incubation Temperature on Fungal Isolation

**Figure 4:**
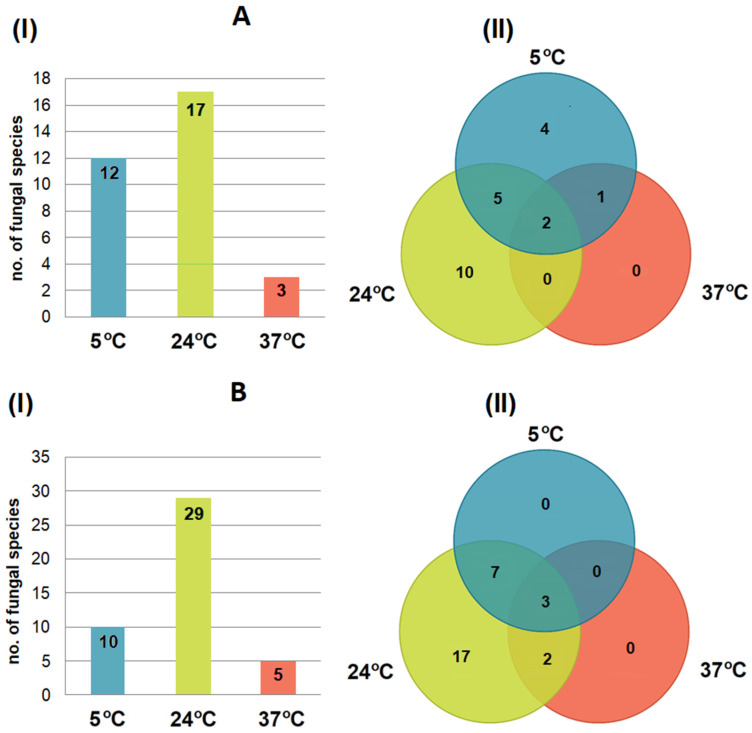
The influence of isolation temperature on the number of cultured fungal species from the wing and tail membranes of *M. myotis* in the underground tunnels of the Nietoperek bat reserve during hibernation (**A**) and in maternity roosts in the attic of the forester’s lodge in Lipy (**B**): (I) the number of species obtained at a given temperature, and (II) the relationships between incubation temperatures and isolated species.

The number of cultured fungal species varied depending on the incubation temperature (Figure 4). The highest species richness was recorded at 24 °C at both study sites (17 and 29 species for hibernation and maternity roosts, respectively), while the lowest was at 37 °C (5 and 3 species for hibernation and maternity roosts, respectively) (Figure 4). Some species were obtained at all three incubation temperatures used (2 and 3 species for hibernation and maternity roosts, respectively), whereas others were temperature-specific (e.g., 10 and 17 species were only obtained at 24 °C for hibernation and maternity roosts, respectively) (Figure 4).

### 3.5. Dominant Fungal Species and the Effect of Biometric Features on Fungal Diversity

**Figure 5:**
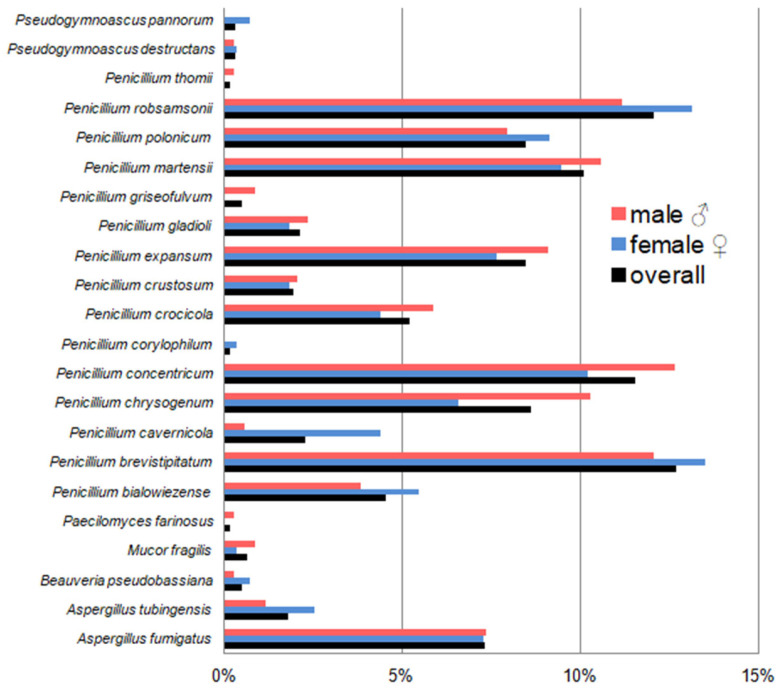
The percentage contribution of each fungal species isolated from the wing and tail membranes of *M. myotis* to the total number of isolates in the underground tunnels of the Nietoperek bat reserve during hibernation.

**Figure 6:**
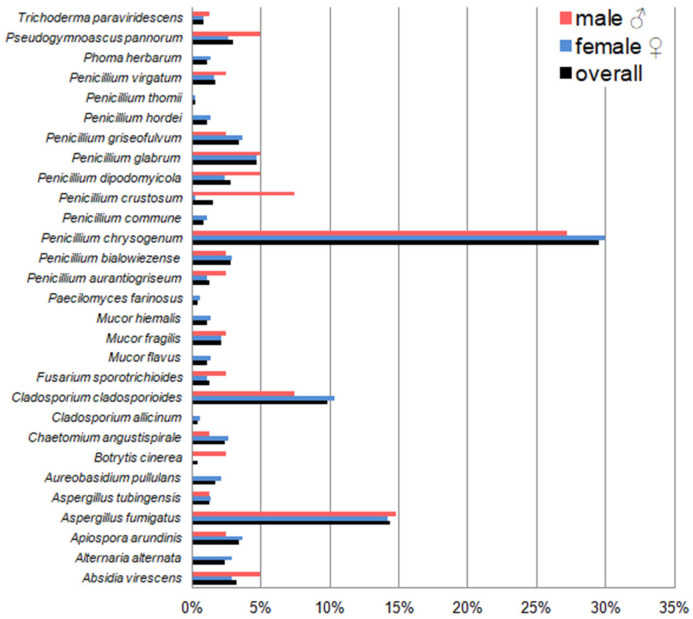
The percentage contribution of each fungal species isolated from the wing and tail membranes of *M. myotis* to the total number of isolates in maternity roosts in the attic of the forester’s lodge in Lipy.

Overall, fungi belonging to the genus *Penicillium* dominated in these studies, constituting almost 90% of all fungi identified in the case of bats hibernating in the underground tunnels of the Nietoperek bat reserve and about 50% in the case of individuals in maternity roosts in the attic of the forester’s lodge in Lipy (Figure 5 and Figure 6, Table A2 and Table A3). In turn, the most frequently isolated fungal species of all the bats studied, regardless of sex and only females during hibernation, was *Penicillium brevistipitatum*, which accounted for 12.70% and 13.50% of all isolated fungi, respectively. In the case of males from this study location, the dominant fungal species was *Penicillium concentricum*—it constituted 12.65% of all isolated fungi (Figure 5, Table A2). On the other hand, the dominant species on the skin *M. myotis* in maternity roosts (without division into sex and with division into female and male) was also *Penicillium chrysogenum*, which accounted for 29.49%, 29.97%, and 27.16% of all obtained species, respectively (Figure 6, Table A3).

The ventral sides of the wing membranes of *M. myotis* were most abundantly colonized by fungi in the Nietoperek bat reserve and in maternity roosts, constituting 28.01% and 29.62%, respectively. In turn, the least fungi were cultured from swabs taken from the ventral sides of the tail membrane of these small mammals at both study sites, constituting 20.36% and 20.80%, respectively (Table A2 and Table A3). In the case of hibernation, *P. chrysogenum* and *Penicillium robsamsonii* dominated on the ventral sides of the wing membranes; *P. brevistipitatum* on the ventral dorsal sides of the wing membranes and the dorsal sides of the tail membrane; and *P. concentricum* on the ventral sides of the tail membrane (Table A2). On the other hand, *P. chrysogenum* was the most frequently isolated species on all examined bat membranes from maternity roosts (Table A3).

*Aspergillus fumigatus*, *Aspergillus tubingensis*, *Mucor fragilis*, *Penicillium bialowiezense*, *P. chrysogenum*, *Penicillium crustosum*, *Penicillium griseofulvum*, *Penicillium thomii*, *Pseudogymnoascus pannorum*, and *Paecilomyces farinosus* were isolated at both study sites (Figure 5 and Figure 6, Table A2 and Table A3). In turn, *Beauveria pseudobassiana*, *P. brevistipitatum*, *Penicillium cavernicola*, *P. concentricum*, *Penicillium corylophilum*, *Penicillium crocicola*, *Penicillium expansum*, *Penicillium gladioli*, *Penicillium martensii*, *Penicillium polonicum*, *P. robsamsonii*, and *Pseudogymnoascus destructans* were cultured only from swabs taken from *M. myotis* during hibernation (Figure 5, Table A2), and *Absidia virescens*, *Alternaria alternata*, *Apiospora arundinis*, *Aureobasidium pullulans*, *Botrytis cinerea*, *Chaetomium angustispirale*, *Cladosporium allicinum*, *Cladosporium cladosporioides*, *Fusarium sporotrichioides*, *Mucor flavus*, *Mucor hiemalis*, *Penicillium aurantiogriseum*, *Penicillium commune*, *Penicillium dipodomyicola*, *Penicillium glabrum*, *Penicillium hordei*, *Penicillium virgatum*, *Phoma herbarum*, and *Trichoderma paraviridescens* were obtained only from samples in maternity roosts (Figure 6, Table A3).

The number of fungal species isolated from bats in both studied habitats tended to decrease with age; however, these relationships were weak and statistically non-significant in both hibernation and maternity roosts (Appendix B).

Similarly, correlations between fungal species richness and other examined bat traits (sex, body mass, and forearm length) were generally weak (r_S_ < 0.3) and not statistically significant across seasons (Appendix B).

A few moderate correlation coefficients were observed (e.g., between forearm length and fungal species richness in males from maternity roosts); however, these patterns were not supported statistically and should therefore be interpreted with caution (full statistical data in Appendix B).

## 4. Discussion

To our knowledge, this is the first study to directly compare the culturable skin mycobiota of *Myotis myotis* between hibernation and reproductive season. The closest comparison comes from previous bacterial studies, where the seasonal turnover of skin microbiota was shown in *Eptesicus fuscus*, with an almost-complete replacement of taxa between summer and winter despite functional redundancy [36]. Although this involved a different host species, both *E. fuscus* and *M. myotis* change roosting habitats seasonally, indicating that similar environmental processes might shape microbial communities in *M. myotis*. However, Grisnik and Walker [36] noted that their sampling was geographically unbalanced, with winter bats collected across a wider range of ecoregions, which may have contributed to the observed differences. For that reason, we examined two colonies located in close proximity, both of anthropogenic origin, where field observations indicate that bats hibernating in Nietoperek frequently form maternity colonies in Lipy, suggesting that the colonies are ecologically linked.

In total, we recovered 22 fungal species during hibernation and 29 during the reproductive season. Previous culture-based surveys of *M. myotis* have been restricted to winter or spring emergence, with 32 airborne species documented in the Nietoperek hibernaculum during winter [10] and 17 species reported from females leaving the same site in spring [9]. Our results align with recent findings from the autumn season, where 39 fungal species were isolated from the wing and tail membranes of *M. myotis* during swarming [7]. Both studies therefore indicate clear seasonal restructuring of the skin mycobiota, with autumn and summer representing periods of high fungal accumulation, while winter shows a much narrower species spectrum.

Culture-based surveys of hibernating *Myotis lucifugus* and *M. septentrionalis* in Canada reported 117 fungal taxa in one survey and 80 in another, with individual bats carrying on average 7–9 species [37,38]. However, comparable culture-based data from the summer season are scarce. In the Neotropics, a survey of insectivorous bats recovered 16 fungal species from the rostral region, with opportunistic taxa such as *Aspergillus sydowii* and *P. crustosum* dominating unidentified *Myotis* spp. individuals [39]. While informative, these findings reflect tropical conditions and cannot be directly compared to European bats’ maternity colonies. Thus, it can be concluded that, to date, culture-based summer data for *M. myotis* remain very limited, and our study helps address this gap.

The higher diversity recorded in summer roosts likely reflects the combined influence of roost microclimate and increased environmental exposure of the bats. Maternity colonies are warmer and more variable than underground hibernacula. Such conditions have been shown to support higher concentrations and the diversity of mesophilic fungi in cave environments [40,41,42]. In addition, active bats forage daily and thus encounter multiple environmental sources of spores, including soil, vegetation, and insect prey, which can subsequently colonize the skin surface [9,39,43]. Several of the taxa isolated in our summer studies, such as *B. cinerea*, *A. alternata*, and *Ph. herbarum*, are typically associated with plants [44,45,46], which are abundant and actively flowering during this period, providing additional reservoirs of fungal propagules. In addition to the external influx of spores, maternity roosts also host dense aggregations of females and young, which may facilitate the internal transmission of environmentally acquired fungi through frequent physical contact [47,48]. Together, these factors provide a plausible explanation for the higher fungal species richness observed during the reproductive season.

In contrast, hibernation sites are cold, humid, and microclimatically stable environments that exert strong selective pressures on the fungal communities [13,49]. Such conditions favour psychrophilic and psychrotolerant taxa, which were also prominent in our winter samples, including *Ps. destructans*, *Ps. pannorum*, and other cold-adapted fungi such as *B. pseudobassiana* and *P. farinosus* [50,51,52].

The recorded ranges of microclimatic conditions during our sampling are consistent with the previous observations at both hibernation sites [53] and in maternity colonies [54]; however, they do not reflect the variation in ambient temperature and relative humidity in both types of bat roosts. The lack of *Pd* on the wing membranes of individuals in maternity roosts in Lipy may be explained by the large differences in microclimatic conditions at hibernation sites (8.9–9.7 °C and 81–84%) and maternity roosts (20–25 °C and 20–40%).

Comparable culture-based surveys of hibernating *Myotis* spp. in North America recovered high numbers of cold-adapted fungi, with *Pseudogymnoascus*, *Oidiodendron*, and *Naganishia* spp. consistently among the most frequent isolates [37,38]. Aeromycological monitoring in caves and mines has likewise shown that winter speleomycobiota are dominated by a smaller set of psychrotolerant propagules [55,56]. Additionally, the absence of foraging during bats’ hibernation further reduces opportunities for acquiring transient environmental fungi, leading to a less diverse but more specialized winter mycobiota, as observed in our samples.

Such specialization was reflected in the dominance of *Penicillium* spp., which accounted for over 90% of isolates in winter compared to roughly 50% of the summer dataset. Many species of this genus can germinate and grow at temperatures close to 0 °C [57]. In vitro studies further demonstrated that *Penicillium* spp. respond to cold by enhancing antioxidant defences and accumulating protective carbohydrates such as trehalose and glycogen [58,59].

However, despite seasonal differences, several taxa overlapped between hibernation and maternity roosts. These included *A. fumigatus*, *A. tubingensis*, *M. fragilis*, and multiple *Penicillium* species, including *P. bialowiezense* and *P. chrysogenum,* as well as *P. farinosus* and *Ps. pannorum*. Notably, members of the family Aspergillaceae, which include the genera *Aspergillus* and *Penicillium*, are among the most cosmopolitan fungi, occurring in diverse climates and habitats ranging from soils and compost to built environments and even arctic substrates [60,61,62]. Even psychrotolerant taxa such as *Ps. pannorum* show broad ecological distribution, having been isolated from Antarctic soils [63], hibernating bats [37], and occasionally from humans where it can act as an opportunistic pathogen despite having a body temperature of ~36.6 °C [64]. In our studies, the occurrence of such fungi across different seasonal samples suggests that a limited group of fungi can persist on *M. myotis* independently of external conditions. Similar observations have been made for bacterial skin microbiota of *E. fuscus*, where seasonal turnover is tempered by the persistence of a few overlapping taxa across summer and winter [36].

When considering individual host traits, biometric variables such as sex, age, body mass, and forearm length showed, at best, weak and inconsistent relationships with fungal richness. For example, fungal richness was higher on wing membranes than on tail membranes, likely reflecting micro-environmental differences such as moisture, temperature, and skin structure, as well as the effects of flight aerodynamics and metabolic heat that make the wings more exposed to spore deposition [65]. However, it can be concluded that morphological traits are not strong predictors of skin mycobiota composition in *M. myotis*. Ecologically, this is not unexpected: while body size or sex may influence contact rates or grooming behaviour to some extent, the dominant drivers of fungal communities appear to be seasonal microclimate and environmental exposure. Thus, biometric traits may modulate fungal richness only marginally, overshadowed by the much stronger seasonal filtering processes. However, these patterns differ from those observed in autumn [7], when age and sex had a stronger influence on fungal richness, including a clear increase in species number with age in males and the opposite trend in females. This suggests that the effect of host traits on the skin mycobiota may change seasonally, being more visible during periods of high environmental exposure and largely reduced during hibernation.

Beyond such seasonal patterns, our study also expands the list of fungi documented from the skin of *M. myotis*. Some taxa obtained here are well established in *M. myotis*-associated mycobiota, whereas others have only been reported from environmental contexts, such as guano, cave air, or cave soil/sediments [7,9,10,14,37,38,49,56,60,66,67,68,69,70,71,72,73,74,75,76,77,78,79,80,81,82,83,84] (Table S1). Several species appear to represent new records for the skin of *M. myotis*, thereby broadening the known ecological range of these fungi. To better contextualize our findings, we summarized previous records of the fungi isolated here in relation to *M. myotis* or other bat species and their environments [7,9,10,14,37,38,49,56,60,66,67,68,69,70,71,72,73,74,75,76,77,78,79,80,81,82,83,84] (Table S1). In total, 27 of the 41 fungal species documented here represent new records for *M. myotis*, while 15 represent new records for bat species in general, as well as 7 that were never reported in bat-associated environments, such as caves. This highlights the extent to which the skin of *M. myotis* may serve as an overlooked reservoir for diverse fungi, bridging environmental, but also pathogenic, taxa.

Our dataset illustrates this pathogenic component, with several opportunistic fungi of medical, agricultural, and veterinary relevance. Among them, *A. fumigatus* is particularly noteworthy due to its clinical relevance as a major cause of aspergillosis in humans [85]. Other taxa frequently detected in summer, such as *A. alternata* and *B. cinerea*, are well-recognized plant pathogens with allergenic potential for humans [44,46]. In winter samples, we detected *Ps. destructans*, the etiological agent of white-nose syndrome, underscoring that the skin of *M. myotis* not only hosts opportunistic fungi but also pathogens with profound impacts on other bat species populations [86]. Together, these findings indicate that the skin of *M. myotis* can also serve as a reservoir for fungi, including the taxa of medical, agricultural, and veterinary importance.

However, in interpreting the results, several limitations should be considered. First, fungal isolation was performed using only one culture medium (PDA). Although this medium is commonly used in mycological studies, it may preferentially promote the growth of fast-growing filamentous fungi, while underrepresenting slow-growing or substrate-specific species [87]. As a result, the fungal diversity reported here likely reflects only the culturable fraction detectable under these laboratory conditions. Second, bats were sampled only once in each season, in contrast to the studies described, e.g., by Kokurewicz et al. [84], who examined airborne fungal composition and changes during the bat hibernation season. Therefore, our design does not allow assessment of short-term temporal fluctuations in the skin mycobiota or potential effects of changing environmental conditions within a given season. These methodological constraints may have influenced the observed patterns. Nevertheless, we believe our study contributes new knowledge regarding the interactions of bats with their skin fungi.

## 5. Conclusions

This study provides the first direct comparison of culturable skin mycobiota in *Myotis myotis* across hibernation and the reproductive season. We found clear seasonal differences: winter assemblages were less diverse but strongly dominated by *Penicillium*, while summer maternity roosts supported broader communities shaped by environmental exposure and plant-associated fungi. A limited set of taxa, including cosmopolitan *Aspergillus fumigatus*, *Mucor fragilis*, and *Pseudogymnoascus pannorum*, persisted across both seasons, indicating the presence of a small core *M. myotis* mycobiota. Fungal richness was highest on wing membranes compared to tail membranes, whereas biometric traits such as sex, age, body mass, and forearm length showed inconsistent associations with diversity. In total, 27 species represent new records for *M. myotis*, underscoring the role of this host as a reservoir that bridges environmental, opportunistic, and pathogenic fungi. These results demonstrate the ecological importance of seasonal filtering in shaping *M. myotis*-associated fungal communities and emphasize the need for further work to explore their functional roles and potential health implications.

## Figures and Tables

**Table 1:** Results of the BLAST analyses of fungi cultured from the wing and tail membranes of *M. myotis* bats in the underground tunnels of the Nietoperek bat reserve during hibernation (isolates UWR_744-UWR_765) and in maternity roosts in the attic of the forester’s lodge in Lipy (isolates UWR_766-UWR_794). All E-values were zero. Sequences were obtained using the primer pairs ITS1 and ITS4 * or/and Bt2a_F and Bt2a_R **.

Isolate Number	Identified Fungi	GenBank Accession No.	The Sequence Length (bp)	Identity with Sequence from GenBank		
				Query Cover (%)	Identity (%)	Accession
UWR_744	*Aspergillus fumigatus*	PX393922 *	522 *	100% *	100% *	MN588001.1 *
UWR_745	*Aspergillus tubingensis*	PX393923 */PX502280 **	403 */388 **	100% */100% **	99.50% */99.23% **	HQ262499.1 */KU711869.1 **
UWR_746	*Beauveria pseudobassiana*	PX393924	390	100%	100%	OR544477.1
UWR_747	*Mucor fragilis*	PX393925	412	100%	100%	PV801950.1
UWR_748	*Paecilomyces farinosus* (syn. *Cordyceps farinosa*)	PX393926	448	100%	100%	AF368793.1
UWR_749	*Penicillium bialowiezense*	PX393927/PX502281	459/381	100%/100%	100/99.48%	OK094894.1/PP524992.1
UWR_750	*Penicillium brevistipitatum*	PX393928	357	100%	100%	MW534763.1
UWR_751	*Penicillium cavernicola*	PX393929/PX502282	386/370	100%/100%	99.22%/100%	NR_163684.1/KJ834439.1
UWR_752	*Penicillium chrysogenum*	PX393930	416	100%	99.76%	MK690561.1
UWR_753	*Penicillium concentricum*	PX393931/PX502283	483/388	100%/100%	100%/98.20%	PV871543.1/OR217443.1
UWR_754	*Penicillium corylophilum*	PX393932/PX502284	503/381	100%/100%	100%/99.48%	JN986758.1/MK450958.1
UWR_755	*Penicillium crocicola*	PX393933/PX502285	459/361	100%/100%	100%/100%	JX869556.1/KU516393.1
UWR_756	*Penicillium crustosum*	PX393934	413	100%	100%	PV935558.1
UWR_757	*Penicillium expansum*	PX393935/PX502286	480/372	100%/100%	100%/98.92%	MN587988.1/MT387277.1
UWR_758	*Penicillium gladioli*	PX393936	404	100%	100%	PV688793.1
UWR_759	*Penicillium griseofulvum*	PX393937/PX502287	504/373	100%/100%	100%/99.73%	KR135143.1/MW080344.1
UWR_760	*Penicillium martensii*	PX393938	451	100%	100%	MH865218.1
UWR_761	*Penicillium polonicum*	PX393939	378	100%	100%	OM892855.1
UWR_762	*Penicillium robsamsonii*	PX393940	374	100%	100%	NR_144866.1
UWR_763	*Penicillium thomii*	PX393941	503	100%	100%	OM415949.1
UWR_764	*Pseudogymnoascus destructans*	PX393942	421	100%	100%	MT015949.1
UWR_765	*Pseudogymnoascus pannorum*	PX393943	394	100%	100%	MT072091.1
UWR_766	*Absidia virescens*	PX393944	484	100%	100%	MZ354150.1
UWR_767	*Alternaria alternata*	PX393945	474	100%	100%	PP781340.1
UWR_768	*Apiospora arundinis*	PX393946	429	100%	99.53%	KX778673.1
UWR_769	*Aspergillus fumigatus*	PX393947/PX502288	415/483	100%/100%	100%/100%	OL589185.1/MN637746.1
UWR_770	*Aspergillus tubingensis*	PX393948	502	100%	100%	PP718796.1
UWR_771	*Aureobasidium pullulans*	PX393949	366	100%	100%	PQ849090.1
UWR_772	*Botrytis cinerea*	PX393950	367	100%	99.18%	OQ625843.1
UWR_773	*Chaetomium angustispirale*	PX393951	368	100%	99.73%	JN209862.1
UWR_774	*Cladosporium allicinum*	PX393952	358	100%	99.44%	OK445637.1
UWR_775	*Cladosporium cladosporioides*	PX393953	481	100%	100%	KM816685.1
UWR_777	*Fusarium sporotrichioides*	PX393955	374	100%	100%	PQ340452.1
UWR_778	*Mucor flavus*	PX393956	400	99%	99.75%	NR_103633.1
UWR_779	*Mucor fragilis*	PX393957	469	100%	100%	PP956644.1
UWR_780	*Mucor hiemalis*	PX393958	457	100%	100%	MN817788.1
UWR_776	*Paecilomyces farinosus* (syn. *Cordyceps farinose*)	PX393954	488	100%	100%	PQ678803.1
UWR_781	*Penicillium aurantiogriseum*	PX393959	498	100%	100%	MZ157166.1
UWR_782	*Penicillium bialowiezense*	PX393960	529	100%	100%	OK510276.1
UWR_783	*Penicillium chrysogenum*	PX393961/PX502289	425/391	100%/100%	100%/96.93%	PQ329214.1/KP329944.1
UWR_784	*Penicillium commune*	PX393962	372	100%	100%	KF990135.1
UWR_785	*Penicillium crustosum*	PX393963	506	100%	100%	KY558627.1
UWR_786	*Penicillium dipodomyicola*	PX393964	468	100%	100%	DQ339570.1
UWR_787	*Penicillium glabrum*	PX393965	388	100%	100%	MK761053.1
UWR_788	*Penicillium griseofulvum*	PX393966	503	100%	100%	PV240451.1
UWR_789	*Penicillium hordei*	PX393967	420	100%	100%	OR513086.1
UWR_790	*Penicillium thomii*	PX393968	499	100%	100%	MZ423029.1
UWR_791	*Penicillium virgatum*	PX393969	389	100%	100%	KF578441.1
UWR_792	*Phoma herbarum*	PX393970	455	100%	100%	KP794136.1
UWR_793	*Pseudogymnoascus pannorum*	PX393971	465	100%	100%	MW113278.1
UWR_794	*Trichoderma paraviridescens*	PX393972	507	100%	100%	KJ728696.1

## Data Availability

Experimental data will be made available upon request. The fungal ITS rDNA nucleotide sequences obtained in the study were submitted to GenBank under accession numbers PX393922-PX393972, and the β-tubulin nucleotide sequences under accession numbers PX502280-PX502289.

## Supplementary Materials

The following supporting information can be downloaded at https://www.mdpi.com/article/10.3390/pathogens15010083/s1, Table S1: Literature records of fungi isolated in this study in relation to *Myotis myotis*.

## Appendix A

**Table A1:** Biometric characteristics of *M. myotis* bats collected from the underground tunnels of the Nietoperek bat reserve during hibernation and from maternity roosts in the attic of the forester’s lodge in Lipy.

Study Sites	Bat No.	Sex	Forearm Length (mm)	Weight (g)	Age
Nietoperek bat reserve	1	male	59.6	24.0	adult
	2	male	59.3	25.0	adult
	3	male	60.5	26.5	subadult
	4	female	62.9	25.0	subadult
	5	male	60.7	23.0	subadult
	6	male	60.3	26.0	adult
	7	male	61.3	24.0	subadult
	8	male	59.1	25.0	subadult
	9	male	59.2	24.5	adult
	10	male	59.9	24.0	adult
	11	female	60.7	26.0	adult
	12	male	61.0	28.0	adult
	13	female	64.1	27.5	adult
	14	male	59.6	23.5	subadult
	15	female	62.7	22.0	adult
	16	male	59.4	24.5	subadult
	17	female	61.0	24.0	adult
	18	female	61.4	27.0	subadult
	19	female	63.2	23.0	subadult
	20	male	61.4	26.0	adult
	21	female	62.0	25.0	adult
	22	male	60.6	24.5	adult
	23	female	63.8	27.5	adult
	24	female	60.6	25.0	adult
	25	male	61.1	24.0	adult
	26	male	58.7	24.0	adult
	27	female	60.5	22.5	subadult
	28	male	60.5	23.0	subadult
	29	female	62.1	26.0	adult
	30	female	61.4	26.0	subadult
forester’s lodge in Lipy	1	female	59.4	26.5	subadult
	2	female	62.5	23.5	subadult
	3	female	61.1	21.0	adult
	4	female	60.8	22.5	subadult
	5	female	63.4	23.5	subadult
	6	female	63.5	24.5	subadult
	7	male	59.9	20.0	subadult
	8	female	63.7	23.0	subadult
	9	female	62.2	27.0	subadult
	10	female	64.8	25.5	subadult
	11	female	63.2	28.0	subadult
	12	female	61.8	23.5	subadult
	13	female	62.5	24.0	subadult
	14	female	63.0	25.0	subadult
	15	female	60.0	29.0	subadult
	16	male	58.1	24.5	subadult
	17	female	60.7	23.5	subadult
	18	female	61.6	24.5	subadult
	19	male	60.0	22.5	subadult
	20	female	62.6	25.0	subadult
	21	female	62.0	26.0	subadult
	22	female	62.2	25.5	subadult
	23	female	61.6	26.5	subadult
	24	female	62.4	24.5	subadult
	25	female	63.9	27.0	subadult
	26	male	56.8	22.5	subadult
	27	female	62.4	23.5	adult
	28	male	59.6	24.0	subadult
	29	female	61.9	24.5	subadult
	30	female	62.5	26.0	subadult

**Table A2:** Diversity of fungal species cultured from the ventral (a) and dorsal (b) sides of the wing membranes (plagiopatagium, dactylopatagia, and propatagium regions), and the ventral (c) and dorsal (d) sides of tail membranes of *M. myotis* (*n* = 30) in the underground tunnels of the Nietoperek bat reserve during hibernation: n.d.—not detected.

Fungal Species	Bat Number and Swab Location		
	5 °C	24 °C	37 °C
*Aspergillus fumigatus*	12 ^(a)^, 18 ^(a,b)^	n.d.	1 ^(c,d)^, 2 ^(a)^, 3 ^(b)^, 4 ^(b,d)^, 5 ^(a,b,c,d)^, 8 ^(a,c)^, 9 ^(d)^, 10 ^(c,d)^, 11 ^(b)^, 12 ^(a,b)^, 14 ^(a,b)^, 15 ^(a,d)^, 16 ^(a)^, 18 ^(a,c,d)^, 19 ^(b,c)^, 20 ^(c,d)^, 21 ^(d)^, 22 ^(c)^, 23 ^(b)^, 24 ^(a,d)^, 25 ^(d)^, 26 ^(b,c)^, 27 ^(c)^, 29 ^(b)^, 30 ^(a,c)^
*Aspergillus tubingensis*	n.d.	15 ^(a)^, 18 ^(c)^	1 ^(c)^, 5 ^(a)^, 8 ^(a)^, 16 ^(a)^, 18 ^(c,d)^, 27 ^(c)^, 29 ^(b)^, 30 ^(a)^
*Beauveria pseudobassiana*	n.d.	1 ^(a)^, 19 ^(a,b)^	n.d.
*Mucor fragilis*	n.d.	16 ^(b)^, 28 ^(a,b)^, 29 ^(a)^	n.d.
*Paecilomyces farinosus*	n.d.	12 ^(d)^	n.d.
*Penicillium bialowiezense*	n.d.	1 ^(a,d)^, 4 ^(a,c)^, 7 ^(b)^, 8 ^(c)^, 14 ^(b,d)^, 19 ^(b)^, 20 ^(b,d)^, 21 ^(a,b,c)^, 22 ^(a,b,d)^, 23 ^(c)^, 24 ^(a,b)^, 25 ^(b)^, 26 ^(d)^, 27 ^(a,b,c)^, 29 ^(d)^, 30 ^(a,c)^	n.d.
*Penicillium brevistipitatum*	2 ^(a,b)^, 6 ^(c,d)^, 10 ^(d)^, 11 ^(b)^, 15 ^(a)^, 19 ^(c)^, 20 ^(b)^, 30 ^(c)^	1 ^(a,b,c,d)^, 3 ^(a,b,d)^, 4 ^(a,b,c)^, 5 ^(a,b,d)^, 6 ^(b,d)^, 7 ^(a,b,d)^, 8 ^(d)^, 9 ^(b,d)^, 10 ^(d)^, 12 ^(b)^, 13 ^(a,b,c,d)^, 14 ^(b,d)^, 15 ^(a)^, 16 ^(a,b)^, 17 ^(a,b,c,d)^, 18 ^(a,b,c,d)^, 19 ^(a,b,c)^, 20 ^(d)^, 21 ^(a,b,c,d)^, 22 ^(a,d)^, 23 ^(a,b,d)^, 24 ^(c)^, 25 ^(a,c,d)^, 26 ^(b)^, 27 ^(b)^, 28 ^(a,b,c,d)^, 29 ^(b,d)^, 30 ^(b,c,d)^	n.d.
*Penicillium cavernicola*	n.d.	4 ^(a,b)^, 5 ^(b)^, 6 ^(d)^, 13 ^(d)^, 17 ^(a,b,c,d)^, 18 ^(a,b,c,d)^, 30 ^(b)^	n.d.
*Penicillium chrysogenum*	1 ^(c)^, 2 ^(a,b,d)^, 6 ^(a)^, 7 ^(a,c,d)^, 9 ^(b)^, 11 ^(a,b)^, 12 ^(a)^, 14 ^(a)^, 15 ^(a,b)^, 19 ^(c,d)^, 20 ^(a,b,c)^, 27 ^(a,d)^, 30 ^(c)^	2 ^(a)^, 3 ^(a,d)^, 5 ^(a)^, 6 ^(a,d)^, 7 ^(a,b,c,d)^, 8 ^(a,b,c,d)^, 9 ^(a,b)^, 10 ^(d)^, 15 ^(b)^, 18 ^(a)^, 19 ^(a,c)^, 20 ^(a)^, 21 ^(b,c)^, 22 ^(d)^, 25 ^(d)^, 26 ^(a,d)^, 27 ^(a)^	5 ^(a)^, 19 ^(c)^, 30 ^(c)^
*Penicillium concentricum*	1 ^(d)^, 5 ^(d)^, 6 ^(b)^, 8 ^(c)^, 12 ^(b)^, 18 ^(b,c,d)^, 19 ^(a,b,c)^, 21 ^(b)^, 22 ^(c)^, 23 ^(b)^, 25 ^(a,c,d)^, 27 ^(c)^, 29 ^(a,c)^	1 ^(a,c,d)^, 2 ^(c)^, 3 ^(c,da)^, 4 ^(a,c)^, 5 ^(d)^, 7 ^(a,b)^, 8 ^(a,b,c,d)^, 9 ^(a,b,d)^, 10 ^(d)^, 11 ^(b)^, 13 ^(a)^, 14 ^(a,c)^, 15 ^(a,d)^, 16 ^(c,d)^, 17 ^(d)^, 18 ^(b)^, 19 ^(a,b,c)^, 20 ^(a,b)^, 21 ^(a,b,c)^, 22 ^(a,d)^, 24 ^(d)^, 26 ^(a,b,c,d)^, 28 ^(a,b,c,d)^, 29 ^(b)^, 30 ^(b)^	n.d.
*Penicillium corylophilum*	n.d.	18 ^(c)^	n.d.
*Penicillium crocicola*	n.d.	2 ^(a,d)^, 3 ^(a)^, 5 ^(d)^, 6 ^(c,d)^, 7 ^(c,d)^, 8 ^(d)^, 9 ^(b,d)^, 11 ^(b,c,d)^, 12 ^(b,c)^, 15 ^(b,c)^, 16 ^(b,c,d)^, 19 ^(a,c)^, 20 ^(c)^, 25 ^(c)^, 26 ^(a,d)^, 28 ^(b)^, 29 ^(a,b)^, 30 ^(c,d)^	n.d.
*Penicillium crustosum*	1 ^(c)^, 4 ^(a)^, 8 ^(c)^, 10 ^(c)^, 11 ^(c)^, 17 ^(a,c)^, 20 ^(a)^, 21 ^(b)^, 26 ^(b)^, 27 ^(b,c)^	n.d.	n.d.
*Penicillium expansum*	1 ^(a)^, 2 ^(a,b)^, 3 ^(a,b)^, 5 ^(c,d)^, 6 ^(c,d)^, 7 ^(a,c,d)^, 8 ^(c)^, 9 ^(b,c)^, 10 ^(c,d)^, 12 ^(b)^, 13 ^(a)^, 14 ^(d)^, 15 ^(a,d)^, 16 ^(a)^, 18 ^(a,b)^, 19 ^(c,d)^, 20 ^(b)^, 22 ^(a,c)^, 23 ^(b)^, 24 ^(d)^, 25 ^(b,d)^, 26 ^(a,b,c)^, 27 ^(b)^, 29 ^(a,b,d)^, 30 ^(b,c)^	6 ^(c)^, 24 ^(d)^	9 ^(d)^, 15 ^(d)^, 16 ^(a)^, 19 ^(b)^, 21 ^(d)^, 29 ^(b)^, 30 ^(a)^
*Penicillium gladioli*	n.d.	1 ^(b)^, 2 ^(a,b)^, 3 ^(b)^, 4 ^(b,c)^, 5 ^(b,c)^, 20 ^(a)^, 24 ^(d)^, 26 ^(b)^, 29 ^(a)^, 30 ^(b)^	n.d.
*Penicillium griseofulvum*	20 ^(c)^	2 ^(a,b)^	n.d.
*Penicillium martensii*	1 ^(c,d)^, 3 ^(a,b,c,d)^, 4 ^(a,b,c)^, 5 ^(a,b)^, 6 ^(b)^, 7 ^(a,b)^, 8 ^(a,b,d)^, 9 ^(a,d)^, 12 ^(b)^, 13 ^(a,b,d)^, 14 ^(a,c,d)^, 16 ^(a,b)^, 17 ^(a,d)^, 18 ^(c,d)^, 19 ^(a,b)^, 20 ^(a,d)^, 21 ^(a,b,d)^, 22 ^(a,b,d)^, 23 ^(a,b,d)^, 24 ^(a,b)^, 25 ^(a,b,c,d)^, 26 ^(b)^, 27 ^(a,b)^, 28 ^(a,b,c,d)^, 29 ^(b,d)^, 30 ^(b,d)^	n.d.	n.d.
*Penicillium polonicum*	1 ^(c)^, 2 ^(a)^, 3 ^(a,b,c,d)^, 4 ^(a,c)^, 5 ^(b)^, 7 ^(b)^, 8 ^(a,c,d)^, 9 ^(a)^, 12 ^(d)^, 13 ^(a)^, 14 ^(b,d)^, 16 ^(a)^, 17 ^(a,b,c,d)^, 18 ^(a,d)^, 19 ^(b)^, 20 ^(a,d)^, 21 ^(a,c,d)^, 22 ^(a,b,c)^, 23 ^(a,b,c)^, 24 ^(a,c)^, 26 ^(b)^, 27 ^(a,c)^, 28 ^(a,b,c,d)^, 29 ^(b,d)^, 30 ^(a,b,d)^	2 ^(a)^	n.d.
*Penicillium robsamsonii*	10 ^(d)^, 13 ^(c)^, 20 ^(c)^	1 ^(a,c)^, 3 ^(a,b,c,d)^, 4 ^(a,b,c)^, 5 ^(a,b,c,d)^, 6 ^(b,c)^, 7 ^(a,b,c,d)^, 9 ^(a,b,d)^, 10 ^(a)^, 11 ^(b)^, 13 ^(a,b,c,d)^, 14 ^(b,c,d)^, 17 ^(a,b,d)^, 19 ^(a,b,d)^, 20 ^(a,b,d)^, 21 ^(a,b,c,d)^, 22 ^(a,b,c)^, 23 ^(a,b,c,d)^, 24 ^(a,b,c)^, 25 ^(a,b)^, 26 ^(a,b)^, 27 ^(a,b,c,d)^, 28 ^(a,b,d)^, 29 ^(a,b,d)^, 30 ^(a,b,d)^	n.d.
*Penicillium thomii*		6 ^(a)^	n.d.
*Pseudogymnoascus destructans*	11 ^(d)^, 12 ^(d)^	n.d.	n.d.
*Pseudogymnoascus pannorum*	27 ^(d)^, 30 ^(d)^	n.d.	n.d.

**Table A3:** Diversity of fungal species cultured from the ventral (a) and dorsal (b) sides of the wing membranes (plagiopatagium, dactylopatagia, and propatagium regions), and the ventral (c) and dorsal (d) sides of tail membranes of *M. myotis* (*n* = 30) in maternity roosts in the attic of the forester’s lodge in Lipy: n.d.—not detected.

Fungal Species	Bat Number and Swab Location		
	5 °C	24 °C	37 °C
*Absidia virescens*	n.d.	3 ^(d)^, 9 ^(a,b,d)^, 10 ^(a)^, 12 ^(b)^, 15 ^(a)^, 17 ^(b,c)^, 26 ^(b,c)^, 28 ^(a,c)^, 30 ^(a,b)^	n.d.
*Alternaria alternata*	n.d.	2 ^(a,b,d)^, 10 ^(a,c)^, 24 ^(a,c)^	2 ^(d)^, 10 ^(a)^, 24 ^(c)^, 25 ^(a)^
*Apiospora arundinis*	12 ^(a)^, 13 ^(a,b)^, 15 ^(b,d)^, 17 ^(c)^, 18 ^(c)^, 20 ^(a)^, 25 ^(d)^, 27 ^(c,d)^, 28 ^(a,b)^	13 ^(a,b)^, 27 ^(d)^	n.d.
*Aspergillus fumigatus*	n.d.	1 ^(c)^, 2 ^(b)^, 3 ^(c)^, 6 ^(d)^, 7 ^(c)^, 8 ^(c)^, 10 ^(c,d)^, 11 ^(d)^, 17 ^(a)^	1 ^(a,b,c)^, 2 ^(a,d)^, 3 ^(b)^, 4 ^(a,b)^, 5 ^(a,b,c)^, 6 ^(a,b,d)^, 7 ^(a,b,d)^, 8 ^(c)^, 9 ^(c,d)^, 10 ^(c)^, 11 ^(a,c,d)^, 12 ^(a,d)^, 13 ^(a,b,c)^, 14 ^(a)^, 15 ^(d)^, 16 ^(a,b,d)^, 17 ^(a,b,c,d)^, 18 ^(a,c,d)^, 19 ^(c,d)^, 20 ^(a,b,d)^, 22 ^(a)^, 23 ^(b)^, 24 ^(a,c)^, 26 ^(a,d)^, 27 ^(c,d)^, 28 ^(c)^, 29 ^(a)^, 30 ^(c)^
*Aspergillus tubingensis*	13 ^(c,d)^	3 ^(c)^, 15 ^(a)^, 19 ^(c)^, 23 ^(d)^	7 ^(c)^, 9 ^(a)^
*Aureobasidium pullulans*	1 ^(a)^, 13 ^(b)^, 20 ^(d)^, 21 ^(a,b)^, 22 ^(a)^	21 ^(a,d)^	n.d.
*Botrytis cinerea*	19 ^(b)^	19 ^(b)^	n.d.
*Chaetomium angustispirale*	n.d.	1 ^(b,d)^, 2 ^(b,c)^, 17 ^(a,c)^, 18 ^(b)^, 20 ^(d)^, 23 ^(d)^, 25 ^(d)^, 28 ^(b)^	
*Cladosporium allicinum*	n.d.	20 ^(a,c)^	n.d.
*Cladosporium cladosporioides*	1 ^(b,d)^, 2 ^(a,d)^, 6 ^(a,d)^, 7 ^(b,c)^, 8 ^(c)^, 9 ^(a,b,d)^, 10 ^(a,d)^, 11 ^(d)^, 12 ^(a,b)^, 15 ^(a,b)^, 17 ^(a,b,d)^, 18 ^(b)^, 19 ^(b)^, 22 ^(a,d)^, 24 ^(a)^, 28 ^(a)^	1 ^(d)^, 2 ^(a,d)^, 6 ^(d)^, 7 ^(b,c)^, 9 ^(a,b)^, 10 ^(a,d)^, 12 ^(a,b)^, 15 ^(a,b)^, 17 ^(a,b)^, 22 ^(a,d)^	n.d.
*Fusarium sporotrichioides*	n.d.	9 ^(a,b,c,d)^, 19 ^(b,d)^	n.d.
*Mucor flavus*	10 ^(b)^	10 ^(a,b)^, 23 ^(a,b)^	n.d.
*Mucor fragilis*	n.d.	2 ^(b)^, 3 ^(b)^, 4 ^(d)^, 5 ^(a)^, 6 ^(d)^, 7 ^(a,c)^, 23 ^(b)^, 29 ^(b,d)^	n.d.
*Mucor hiemalis*	16 ^(b,c,d)^, 24 ^(b,c)^	9 ^(a,b)^, 14 ^(b)^, 16 ^(b,c)^, 24 ^(d)^	26 ^(a,b)^, 29 ^(b)^
*Paecilomyces farinosus*	n.d.	13 ^(d)^, 22 ^(d)^	n.d.
*Penicillium aurantiogriseum*	n.d.	19 ^(a,b)^, 30 ^(a,b,c,d)^	n.d.
*Penicillium bialowiezense*	n.d.	1 ^(c,d)^, 2 ^(a,b,d)^, 4 ^(a)^, 6 ^(d)^, 7 ^(a)^, 10 ^(a)^, 12 ^(b)^, 17 ^(b,c)^, 28 ^(b)^	n.d.
*Penicillium chrysogenum*	1 ^(a,b)^, 3 ^(a)^, 4 ^(a,b,c)^, 5 ^(b,c,d)^, 6 ^(b,c,d)^, 7 ^(b,d)^, 8 ^(a,b,c,d)^, 10 ^(c,d)^, 11 ^(a,b)^, 14 ^(a,b,c,d)^, 15 ^(a,b,c)^, 16 ^(c,d)^, 18 ^(a,b,c,d)^, 19 ^(a)^, 20 ^(a,c)^, 21 ^(b)^, 22 ^(a,c,d)^, 23 ^(b)^, 24 ^(c,d)^, 25 ^(a)^, 26 ^(a,d,c)^, 27 ^(a,b,c)^, 28 ^(c)^, 29 ^(a,b,c,d)^, 30 ^(b)^	1 ^(a,c,d)^, 2 ^(a,c)^, 3 ^(b,c)^, 4 ^(a,b,c,d)^, 5 ^(b,c,d)^, 6 ^(a,b,c,d)^, 7 ^(a,b,d)^, 8 ^(a,b,d)^, 9 ^(c,d)^, 10 ^(d)^, 11 ^(a,b,c,d)^, 12 ^(c,d)^, 13 ^(a,b,d)^, 14 ^(a,b,d)^, 15 ^(a,d)^, 16 ^(c,d)^, 17 ^(b,c)^, 18 ^(b,c,d)^, 19 ^(a)^, 20 ^(c)^, 21 ^(a,c)^, 22 ^(b)^, 23 ^(c)^, 24 ^(c,d)^, 25 ^(a,b)^, 26 ^(a,d)^, 27 ^(a,b,c,d)^, 28 ^(a,b,c,d)^, 29 ^(b)^	3 ^(c)^, 4 ^(c)^, 5 ^(a)^, 6 ^(a,c,d)^, 8 ^(b)^, 16 ^(d)^, 17 ^(a,d)^
*Penicillium commune*	n.d.	12 ^(a,b)^, 21 ^(a)^, 23 ^(a)^	n.d.
*Penicillium crustosum*	n.d.	18 ^(b)^, 26 ^(a,b,c,d)^, 28 ^(b,d)^	n.d.
*Penicillium dipodomyicola*	n.d.	6 ^(b)^, 7 ^(b)^, 10 ^(d)^, 11 ^(c,d)^, 14 ^(c)^, 16 ^(a,c,d)^, 20 ^(b)^, 21 ^(b)^, 23 ^(b,c)^	n.d.
*Penicillium glabrum*	n.d.	2 ^(a,d)^, 4 ^(a)^, 8 ^(a)^, 9 ^(a,b)^, 10 ^(a,b,c)^, 16 ^(d)^, 17 ^(b)^, 18 ^(b)^, 19 ^(b)^, 20 ^(a,b)^, 22 ^(a)^, 24 ^(b)^, 25 ^(a)^, 26 ^(c)^, 27 ^(a)^, 28 ^(a)^, 30 ^(a)^	n.d.
*Penicillium griseofulvum*	1 ^(a,d)^, 3 ^(c)^, 7 ^(a)^, 9 ^(d)^, 18 ^(c,d)^, 20 ^(c)^, 23 ^(c,d)^, 26 ^(d)^	16 ^(a)^, 17 ^(d)^, 18 ^(a)^, 20 ^(b)^, 23 ^(b)^, 25 ^c)^,	n.d.
*Penicillium hordei*	n.d.	29 ^(c)^, 30 ^(a,b,c,d)^	n.d.
*Penicillium thomii*	n.d.	30 ^(b)^	n.d.
*Penicillium virgatum*	n.d.	5 ^(a)^, 24 ^(a,d)^, 25 ^(b,c,d)^, 28 ^(a,b)^	n.d.
*Phoma herbarum*	n.d.	1 ^(b,c)^, 2 ^(d)^, 5 ^(a)^, 24 ^(a)^	n.d.
*Pseudogymnoascus pannorum*	6 ^(a)^, 7 ^(b)^, 8 ^(a)^, 13 ^(a)^, 20 ^(a)^, 26 ^(a,b)^	6 ^(a)^, 7 ^(c)^, 12 ^(b)^, 15 ^(a)^, 20 ^(a)^, 21 ^(a)^, 29 ^(c)^	n.d.
*Trichoderma paraviridescens*	n.d.	15 ^(b,c,d)^, 19 ^(b)^	n.d.

## Appendix B. Supplementary Correlation Analyses

The number of fungal species inhabiting the bats in both studied habitats decreased with age (r_S_ = −0.32831, *p* = 0.07651 for hibernation; r_s_ = −0.26534, *p* = 0.15645 for maternity roosts). The Spearman (r_S_) values for the interactions between the number of fungal species and the remaining examined bat traits were in the range above −0.2 and below 0.2, suggesting weak correlations, which proves the lack of relationship between the examined data (r_S_ = 0.06333, *p* = 0.73953 for sex in hibernation; r_S_ = −0.04357, *p* = 0.81918 for forearm length during hibernation; r_S_ = 0.05239, *p* = 0.78337 for weight during hibernation; r_S_ = −0.1712, *p* = 0.36571 for forearm length in maternity roosts; r_S_ = 0.04772, *p* = 0.80226 for weight in maternity roosts; r_S_ = −0.11492, *p* = 0.54538 for sex in maternity roosts).

A low positive correlation was noted between the number of fungal species inhabiting *M. myotis* males and their weight during hibernation (r_S_ = 0.23734, *p* = 0.35902), and in the case of summer studies, this relationship had a low negative correlation (r_S_ = −0.20520, *p* = 0.74058), but in both cases, it was not statistically significant. In turn, a moderate positive correlation, but statistically insignificant, was noted between the forearm length and the number of species inhabiting *M. myotis* males in maternity roosts (r_S_ = 0.60000, *p* = 0.28476), while in wintering colonies, this relationship was close to zero (r_S_ = 0.03959, *p* = 0.88008). A similar situation with weak positive and statistically insignificant correlations was found between the age of males during hibernation and the number of fungal species inhabiting them (r_S_ = 0.03872, *p* = 0.88271). In turn, the number of fungal species colonizing females of these small mammals correlated negatively, but statistically insignificantly, to varying degrees, with their biometric traits, both in hibernation (r_S_ = −0.22596, *p* = 0.45791 for forearm length; r_S_ = −0.06629, *p* = 0.82964 for weight; except for r_S_ = −0.66418, *p* = 0.01329 for age) and maternity roosts (r_S_ = −0.19744, *p* = 0.34414 for forearm length; r_S_ = −0.2903, *p* = 0.15922 for age). The exception was the body mass of the male during the summer in the attic of the forester’s lodge in Lipy, which correlated weakly and positively with the number of inhabiting fungal species (r_S_ = 0.17711, *p* = 0.39703), but statistically insignificantly.
