# Supplementary material for: Seasonal Turnover in Bat Skin Mycobiota: Contrasting Fungal Communities Between Hibernation and Reproduction in Greater Mouse-Eared Bats (Myotis myotis)
Source: Pathogens. 2026 Jan 12;15(1):83. doi: 10.3390/pathogens15010083 (PMC12845230; doi:10.3390/pathogens15010083)
Supplement: Supplementary file 1 [file pathogens-15-00083-s001.zip › pathogens-4061693-supplementary.pdf]

Supplementary Material

Table S1. Literature records of fungi isolated in this study in relation to *Myotis myotis*. The table indicates whether each taxon has previously been reported from the skin of *M. myotis*, from other bat-associated environments (e.g., guano, cave air, cave walls), or other bat species. Abbreviations: n.f. – not found; u.a. – unassigned; *Cp – Carollia perspicillata; De – Diphylla ecaudata; Ef – Eptesicus fuscus; Hs – Hypsugo savii; Ma – Myotis austroriparius; Mc – Myotis capaccini; Mdas – Myotis dasycneme; Mdau – Myotis daubentonii; Me – Myotis emarginatus; Mle – Myotis leibii; Mlu – Myotis lucifugus; Mmyo – Myotis myotis; Mmys – Myotis mystanicus; Mn – M. natteri; Mob – Miniopterus orianae bassanii; Moo – Miniopterus orianae oceanensis; Mp – Myotis pilosus; Mso – Myotis sodalis; Mse – Myotis septentrionalis; Msch – Miniopterus schreibersii; Mv – Myotis velifer; Pa – Plectotus auritus; Pn – Pipistrellus nathusii; Ps – Pipistrellus subflavus; Rs – Rhinolophus sinicus; Tt – Tadarida teniotis*.

Fungal species	Reported from *M. myotis* skin	Reported from other bat species' skin	Other observations
Absidia virescens	n.f.	n.f.	n.f.
Alternaria alternata	[7]	n.f.	Cave aeromycota bat guano* [60,66]
Apiospora arundinis	[7]	*Mp* and *Msch* [14]	n.f.
Aspergillus fumigatus	[7,9]	*Mdau, Mlu, Mse, Pn,* and *Tt* [37,38, 67,68]	Cave aeromycota around *Mmyo* [10], cave aeromycota and bat guano [66]
Aspergillus tubingensis	n.f.	n.f.	Cave aeromycota around *Mmyo* [10]
Aureobasidium pullulans	[7]	*Mdau, Mlu, Moo,* and *Mse* [38,68,69]	Cave aeromycota and sediments [37,56]
Beauveria pseudobassiana	n.f.	n.f.	Cave church [70]
Botrytis cinerea	n.f.	*Mdau* and *Pa* [68]	Cave aeromycota [56,60,66] and cave aeromycota around *Mmyo* [10]
Chaetomium angustispirale	n.f.	*Mdau, Mmys* and *Pa* [68]	n.f.
Cladosporium allicinum	[7]	*Mdas, Mdau, Me, Mys,* and *Pa* [68]	Cave soil/sediments [49]
Cladosporium cladosporioides	[7]	*Cp, De, Hs,* and *Mdau* [67,68,71]	Cave aeromycota around *Mmyo* [10]; cave aeromycota, soil/sediments, and dead *Mlu* and *Mse* [37,60, 72]
Fusarium sporotrichioides	n.f.	n.f.	Cave aeromycota [37]
Mucor flavus	n.f.	*Me* [68]	Cave aeromycota around *Mmyo* [10], cave soil/sediments [37] and bat dry guano [73]

Mucor fragilis	n.f.	*Mdau* [68]	Cave aeromycota around *Mmyo* [10] and cave aeromycota [37]
Mucor hiemalis	n.f.	*Mc*, *Msch*, u.a. *Myotis* sp., and u.a. *Pipistrellus* sp. [67]	Cave aeromycota, bat guano, and hibernacula [60,66,68]
Paecilomyces farinosus	[74]	*Mlu* and *Mse* [37,38]	Cave soil/sediments, aeromycota and bat guano [49,60]
Penicillium bialowiezense	[7]	*Mdas*, *Mdau*, *Mn*, *Moo*, and *Pa* [68,69]	Cave aeromycota around *Mmyo* [10]
Penicillium brevistipitatum	[9]	n.f.	Cave aeromycota around *Mmyo* [10] and bat dray guan [73]
Penicillium cavernicola	n.f.	n.f.	Cave aeromycota around *Mmyo* [10]
Penicillium chrysogenum	[9]	*Ef*, *Mlu*, and *Pa* [37,38,68,75]	Cave aeromycota around *Mmyo* [10], cave aeromycota, soil/sediments, wall, bat guano [37,60,66,72,75], water and sediment and bat moist guan [73]
Penicillium concentricum	n.f.	*Mlu* and *Mse* [37,38]	Cave aeromycota around *Mmyo* [10], cave soil/sediments, wall and rodent dung [49,75]
Penicillium commune	n.f.	*Mlu* [38]	Cave aeromycota around *Mmyo* [10], cave aeromycota, soil/sediments, and bat guano [37,60], soil and bat dry guan [73]
Penicillium corylophilum	n.f.	*Mdau* and *Pa* [68]	Cave aeromycota, soil/sediments, wall, and bat guano [37,60,75]
Penicillium crocicola	n.f.	n.f.	n.f.
Penicillium crustosum	n.f.	*Mdau* and *Moo* [68,69]	Cave aeromycota around *Mmyo* [10], cave aeromycota, wall and soil/sediment [37] and bat dry guan [73]
Penicillium dipodomyicola	n.f.	n.f.	n.f.
Penicillium expansum	n.f.	n.f.	Cave aeromycota around *Mmyo* [10], cave aeromycota, wall, soil/sediments and bat guano [37,60,72,75]

Penicillium glabrum	n.f.	*Rs* [14]	Cave aeromycota, soil/sediments and bat guano [37,56,60,75]
Penicillium gladioli	n.f.	n.f.	n.f.
Penicillium griseofulvum	n.f.	*Pipistrellus* sp. [67]	Cave soil/sediments, water, wood, wall, dead bats and bat dry guano [37,73]
Penicillium hordei	n.f.	n.f.	Cave bat guano [37,60]
Penicillium martensii	n.f.	n.f.	n.f.
Penicillium polonicum	[7]	*Mob*, *Mmys*, and *Pa* [68,69]	Cave wall [37] and bat moisdry guan [73]
Penicillium robsamsonii	n.f.	n.f.	Cave aeromycota around *Mmyo* [10]
Penicillium thomii	n.f.	*Mlu* and *Mse* [37,38]	Cave earthworm casts [60,72]
Penicillium virgatum	n.f.	n.f.	n.f.
Phoma herbarum	n.f.	n.f.	n.f.
Pseudogymnoascus destructans	[74]	*Ef*, *Ma*, *Mle*, *Mlu*, *Mse*, *Mso*, *Mv*, and *Ps* [37,38,76]	Cave aeromycota, soil/sediments, and bat guano [77,78,84]
Pseudogymnoascus pannorum	[7]	*Mlu*, *Mse*, and *Ps* [37,38]	Cave aeromycota, soil/sediments, wall and bat guano [37,49,60,72,79-82]
Trichoderma paraviridescens	n.f.	n.f.	Cave bat guano [83]

*In Nováková [60], bat guano was identified as originating from *Miniopterus schreibersii* or *Rhinolophus euryale*; in other studies the bat species producing guano was not specified. Importantly, guano from *M. myotis* was not reported in any of these cases.
